# Ultrasound harmonic generation and atomic layer deposition of multilayer, deep-UV mirrors and filters with microcavity plasma arrays

**DOI:** 10.1140/epjd/s10053-023-00651-3

**Published:** 2023-05-11

**Authors:** Jinhong Kim, Andrey Mironov, Sehyun Park, Changgong Kim, Sung-Jin Park, J. Gary Eden

**Affiliations:** 1 Laboratory for Optical Physics and Engineering, Department of Electrical and Computer Engineering, University of Illinois, 306 N Wright St, Urbana, IL 61801 USA; 2 Department of Mechanical Science and Engineering, University of Illinois, 1206 W Green St, Urbana, IL 61801 USA; 3 Intel Corp., 2501 NE Century Blvd, Hillsboro, OR 97124 USA; 4 Eden Park Illumination, 902 N. Country Fair Drive, Champaign, IL 61820 USA

## Abstract

**Abstract:**

In honor of Professor Kurt Becker’s pioneering contributions to microplasma physics and applications, we report the capabilities of arrays of microcavity plasmas in two emerging and disparate applications. The first of these is the generation of ultrasound radiation in the 20–240 kHz spectral range with microplasmas in either a static or jet configuration. When a $10\times 10$ array of microplasma jets is driven by a 20-kHz sinusoidal voltage, for example, harmonics as high as *m* = 12 are detected and *fractional harmonics* are produced by controlling the spatial symmetry of the emitter array. The preferential emission of ultrasound in an inverted cone having an angle of $\pm \,45^\circ$ with respect to the surface normal of the jet array’s exit face is attributed to interference between spatially periodic, outward-propagating waves generated by the arrays. The spatial distribution of ultrasound generated by the arrays is analogous to the radiation patterns produced by Yagi-Uda phased array antennas at RF frequencies for which radiation is emitted broadside to arrays of parallel electric dipoles. Also, the nonperturbative envelope of the ultrasound harmonic spectrum resembles that for high-order harmonic generation at optical frequencies in rare gas plasmas and attests to the strong nonlinearity provided by the pulsed microplasmas in the sub-250-kHz region. Specifically, the relative intensities of the second and third harmonics exceed that for the fundamental, and a “plateau” region is observed extending from the 5th through the 8th harmonics. A strong plasma nonlinearity appears to be responsible for both the appearance of fractional harmonics and the nonperturbative nature of the acoustic harmonic spectrum. Multilayer metal-oxide optical filters designed to have peak transmission near 222 nm in the deep-UV region of the spectrum have been fabricated by microplasma-assisted atomic layer deposition. Alternating layers of ZrO$_2$ and Al$_2$O$_3$, each having a thickness in the 20–50 nm range, were grown on quartz and silicon substrates by successively exposing the substrate to the Zr or Al precursor (tetrakis(dimethylamino) zirconium or trimethylaluminum, respectively) and the products of an oxygen microplasma while maintaining the substrate temperature at 300 K. Bandpass filters comprising 9 cycles of 30-nm-thick ZrO$_2$/50-nm-thick Al$_2$O$_3$ film pairs transmit 80% at 235 nm but < 35% in the 250–280 nm interval. Such multilayer reflectors appear to be of significant value in several applications, including bandpass filters suppressing long wavelength (240–270 nm) radiation emitted by KrCl (222) lamps.

**Graphical abstract:**

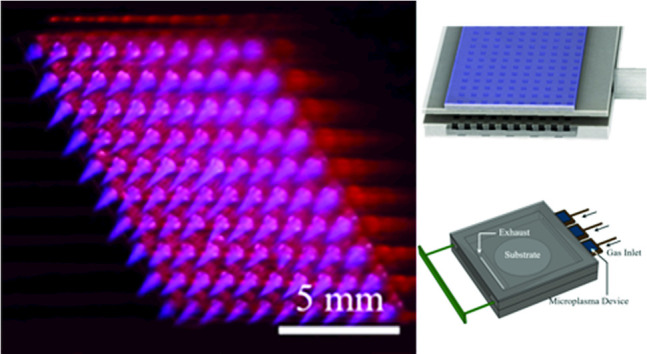

## Introduction

On the occasion of this Special Issue commemorating Professor Becker’s 70th birthday, we are pleased to present the results of two series of experiments which demonstrate the unanticipated ability of microcavity plasma arrays to serve in specific new but promising applications. The emergence of microplasmas in the mid-1990s has culminated in a remarkable variety of applications, of which only a few were envisioned when the field was in its infancy [1, 2]. In the sections to follow, the first results to be presented demonstrate the potential of arrays of microcavity plasmas as compact and tunable generators of ultrasound radiation in the 20–240 kHz frequency interval. Not only are harmonics of the driving field produced (up to $m=12$), but the generation of *half-integer* harmonics has been observed and found to be dependent upon the spatial symmetry (or lack thereof) of the array geometry. Both static and plasma jet configurations are described, the latter of which launches an ultrasound beam in the form of an inverted cone having an angle of 45$^\circ$ (± 20$^\circ$) with respect to the axis of the microjets. The spatial distribution of ultrasound generated by the arrays is analogous to the radiation patterns produced by Yagi-Uda phased array antennas at RF frequencies for which radiation is emitted broadside to arrays of parallel dipoles. Owing to the columns and rows of microjets in each two-dimensional array, spatially orthogonal ultrasound waves are emitted, and interference between them is responsible for off-axis ultrasound propagation. We also report here the fabrication of multilayer dielectric filters and mirrors for the deep-ultraviolet (UV) spectral region by microplasma-assisted atomic layer deposition (MALD). Alternating layers of ZrO$_2$ and Al$_2$O$_3$, each having a thickness in the 20–50 nm range, were grown on quartz and silicon substrates by successively exposing the substrate to the Zr or Al precursor (tetrakis(dimethylamino) zirconium [TDMAZ] or trimethylaluminum [TMA], respectively) and the products of an oxygen microplasma while maintaining the substrate temperature at 300 K. Bandpass filters comprising 7 cycles of 30-nm-thick ZrO$_2$/50 nm-thick Al$_2$O$_3$ film pairs transmit 70% at 232 nm but only 30% in the 255–260-nm interval. The potential of these nascent technologies as compact and phased sources of ultrasound, or for the fabrication of precision optical components, appears to be significant.

## Generation of ultrasound (20–240 kHz) by arrays of microplasmas

The generation of ultrasound has been studied extensively in several contexts but primarily with the intention of developing new sources suitable for nondestructive testing of materials [3–9] or the replacement of conventional transducers for biomedical diagnostics. Pulsed lasers and high-voltage spark or corona discharges have been pursued but both suffer from several drawbacks. Generating ultrasound with pulsed lasers is capable of producing intense ultrasound signals but a “dummy” target and laser intensities of typically $10^7\,\text {W-cm}^{-2}$ are required to form a plasma, thus rendering this approach prohibitively expensive and unsuitable for biomedical applications, for example [6, 7]. In contrast, both corona and spark discharges have been popular with audio enthusiasts for decades because of the high-frequency response, omnidirectionality, and fidelity available with “plasma speakers”. It has long been known that conventional speakers are unable to reproduce, for example, square waves at audio frequencies because of the transducer mass, but plasma speakers dispense with the diaphragm inherent to conventional audio systems [10–12]. Despite the excellent audio reproduction characteristics of corona and spark plasma systems, however, plasma speakers remain the domain of hobbyists to this day. Because of the excessive voltages and bulky components required, but also the dominance of streamers in such discharges, plasma systems have not been competitive with advanced transducers. The advent of arrays of microcavity plasmas alters the landscape of ultrasound generation significantly, primarily because of the ability to produce glow discharges at atmospheric pressure, even in the presence of attaching gases. Furthermore, the ability to generate ultrasound from literally thousands of microcavities, each of which can be controlled separately from all others, provides the opportunity to vary at will the phase of an array of microplasma transducers.

**Fig. 1:**
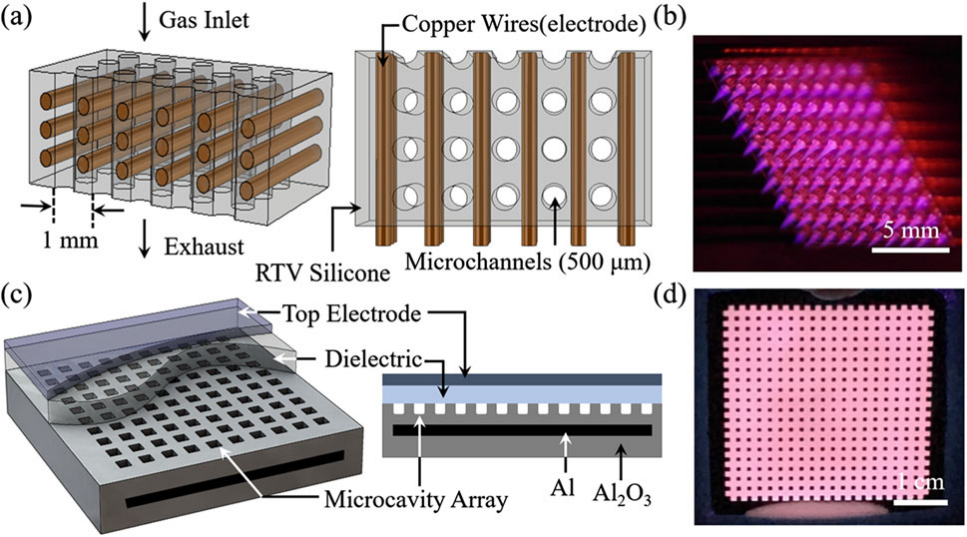
**a, c** Schematic diagrams of the structures for the microplasma jet and static devices. The electrode array for the microjet array comprises metal micro-rods embedded in silicone, and plasma is generated in He flowing in 500-$\upmu$m-diameter microchannels. The static structure is a sealed 10 $\times$ 10 array of microcavities fabricated in nanoporous alumina having an integrated Al lower electrode. The dielectric by which the microcavity array is sealed is either glass or quartz; **b, d** photographs of the microplasma jet and static (sealed) arrays in operation

Schematic diagrams of the jet and static device configurations fabricated at the University of Illinois and adopted for the ultrasound generation experiments are shown in panels (a) and (c) of Fig. 1. Arrays of microplasma jets were produced by flowing a rare gas through cylindrical microchannels formed in a silicone polymer, as illustrated in Fig. 1a. Plasma was generated in each of the 500-$\upmu$m-diameter microchannels with copper rod electrodes, embedded in the polymer and oriented orthogonal to the microchannels. Note that the electrode arrangement of Fig. 1a allows for the generation of plasma in specific channels at will (i.e., addressability). For the experiments discussed here, the electrode array was driven by a 20–50-kHz sinusoidal voltage waveform ($V_\textrm{RMS} = 848$ V) and the flowing feedstock gas was He at a pressure of 740 Torr. A photograph of a 10 $\times$ 10 array of microplasma jets emerging from the silicone block is given in Fig. 1b, and further details regarding the design and operation of the microjet array can be found in Ref. [13]. Panel (c) of Fig. 1 presents a cutaway view of the dielectric barrier discharge (DBD) structure that served as a static ultrasound generator. For these devices, microcavities were laser-micromachined into nanoporous Al$_2$O$_3$ grown from Al foil [14], and a film of indium tin oxide (ITO) covering a thin sheet of quartz or glass (dielectric of Fig. 1c) served as the top electrode to which the driving voltage was applied. As shown by the cross-sectional diagram of Fig. 1c, both the ITO and Al electrodes were common to all of the microcavities in the $10 \times 10$ arrays, but it should be noted that converting the electrode geometry to an array of addressable pads is straightforward. After evacuating this sealed array to 10$^{-3}$ Torr and backfilling with one atmosphere of research grade helium (99.999% purity), visible luminescence is observed to uniformly cover the microplasma grid (cf. Figure 1d).

The relative ultrasound intensity generated by the two types of arrays was measured with a condenser microphone having a 500-kHz sampling rate and a low-noise anti-aliasing filter (210 kHz). Because the vibration of the ITO electrode/dielectric surface of the static device is weak, a Polytec OFV-500 laser vibrometer was employed for the static device tests and the measurements of surface displacement were confirmed with a 40-$\upmu$m-thick membrane placed $\sim$ 3 cm from the static device. Driving voltage frequencies in the 20–50-kHz interval and microplasma jet arrays ranging in size from $1 \times 10$ to $10 \times 10$ were studied for the purpose of examining symmetry effects. In performing such tests, the size of the jet array can be changed simply by addressing the desired number of microchannels in a single 10 $\times$ 10 array.

**Fig. 2:**
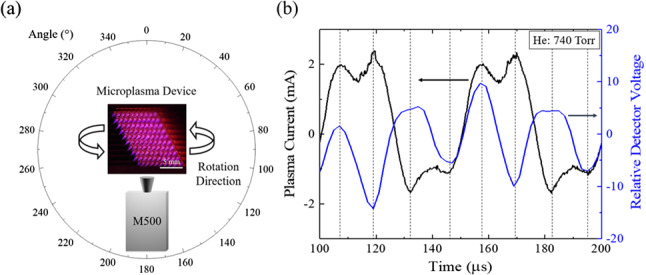
**a** Illustration of the experimental arrangement with which the ultrasound emission intensity of the microplasma jet array is measured by an M500 condenser microphone as the microphone is rotated between $-90^\circ$ and $+90^\circ$ with respect to the surface normal of the array; **b** representative plasma current and microphone voltage waveforms (black and blue, respectively). In acquiring these waveforms, the pressure of the flowing He gas was set at 740 Torr, and multiple measurements were taken to ensure the reproducibility of the results

Figure 2a qualitatively illustrates the arrangement with which the azimuthal symmetry of the emission of ultrasound by the microplasma jet array was measured. The relative emission of the array was first recorded by a condenser microphone (M500) viewing the array at normal incidence to the exit plane of the jet array. With the array position fixed, the microphone was rotated in a goniometer arrangement so as to continuously monitor the relative ultrasound intensity as the observation angle was varied by ± 90$^\circ$ with respect to the surface normal. During these measurements, the radius of the arc through which the detector was rotated was fixed at 3 cm. Representative waveforms of the microjet plasma array current and condenser microphone voltage, recorded for a driving voltage frequency of 20 kHz, are presented in panel (b) of Fig. 2. Note that the detector voltage is modulated at 40 kHz, or twice the driving frequency, which is an initial indication that the microplasma jet array is producing ultrasound harmonics of the driving voltage. It should also be emphasized that, throughout these experiments, multiple measurements were taken for each set of experimental conditions (RMS voltage, polar angle of detector relative to array surface normal, array size, etc.) so as to confirm the reproducibility of the data.

**Fig. 3:**
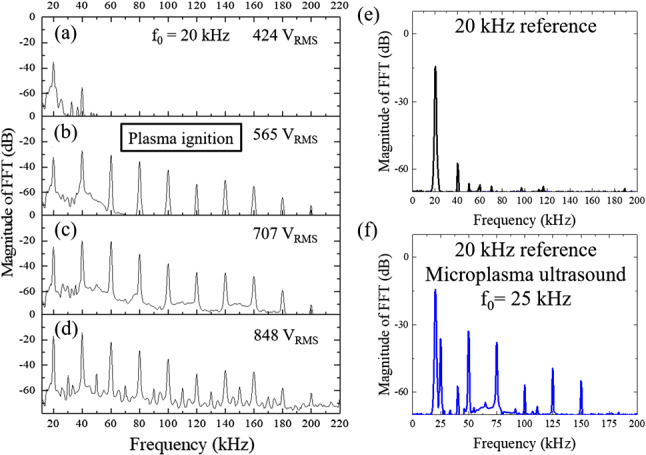
Fourier (FFT) ultrasound spectra recorded for a 10 $\times$ 10 array of microplasma jets, and a piezoelectric speaker: **a–d** dependence of the microplasma jet Fourier spectrum on the array driving voltage. For (**a**), the driving voltage of 424 V (RMS) lies below breakdown which occurs in the vicinity of 565 V$_{RMS}$; **e** reference spectrum provided by a piezoelectric speaker driven at 20 kHz; **f** superposition of the spectrum of (**e**) with that generated by a microplasma jet array when driven at a fundamental frequency of 25 kHz. Note that virtually all of the harmonic structure is produced by the jet array. All of the spectra shown are power spectra in which the ordinate represents the square of the Fourier magnitude, $\vert F(\omega )\vert ^2$

Figure 3 summarizes the results of measurements of the ultrasound spectra generated by a 10 $\times$ 10 microplasma jet array when driven at 20 kHz with RMS voltages ranging from 424 to 848 V. Specifically, the Fourier (spectral) domain representation of the ultrasound detector voltage vividly illustrates the strength of the nonlinearity associated with the collective generation of acoustic waves by the microplasma array. Figure 3a is intentionally recorded for a 20-kHz driving voltage of 424 V (RMS) which lies below electrical breakdown for a $10\times 10$ array of microplasma jets. Consequently, the ultrasonic response of the array is limited to the production of a frequency component at the fundamental frequency ($f_0=20$ kHz) and a weak response (i.e., near the detection limit) at the second harmonic frequency of 40 kHz. Both are attributable to the ionization and slight subsequent heating of the He gas flow that occurs despite the absence of electrical breakdown. When the driving voltage is raised to 565 $V_\textrm{RMS}$, however, the 10 $\times$ 10 array of plasma jets is ignited and a rich harmonic spectrum, extending to at least $m=10$, is observed (Fig. 3b–d). Because of the limited bandwidth of the condenser microphone, the 12th harmonic was the highest observed reproducibly but much higher harmonics are undoubtedly being generated. Study of the full spectrum being produced by the jet array must await the availability of an ultrasound detector of larger bandwidth. Of most significance in Fig. 3b, however, is the Fourier amplitude of the second harmonic (40 kHz) which *exceeds* that for the fundamental. Quite unexpectedly, the amplitude of the 4th harmonic (80 kHz) almost matches the strength of the 2nd harmonic and the amplitudes of still higher harmonics fall monotonically, but slowly, with increasing order number, *m*. As the driving voltage is increased further (707 V$_\textrm{RMS}$ and 848 V$_\textrm{RMS}$ for panels (c) and (d) of Fig. 3, respectively), a continuum first appears and, eventually, fractional harmonics (such as $m=2.5, 3.5, 4.5, \ldots$ at 50, 70, 90 kHz, respectively) are also observed. It must be emphasized that all of the spectral features shown in Fig. 3 are reproducible and not electronic artifacts. Indeed, the joint appearance of the spectral continuum with “half-harmonics”, as well as the appearance of reproducible fractional harmonics, including those at 35, 65, and 95 kHz ($m=1.75, 3.25$, and 4.75), provide additional confirmation of the strength of the *m*th-order nonlinearity that is responsible for the acoustic wave mixing evident in Fig. 3. As discussed later, the appearance of fractional harmonics is evidence of nonlinear wave mixing in the microjet emission.

In an effort to benchmark the relative measurements of Fig. 3, analogous tests were performed with a piezoelectric speaker (DB Unlimited: SZ250808-1) that serves as an ultrasound reference. For a separation of 10 cm (in air) from the piezoelectric speaker to the detector, the average value for the standard pressure level (SPL) produced by the speaker at 1, 1.5, 1.8, 2, 3, and 4 kHz was inferred from manufacturer data and measured detector waveforms to be 89 dBA (where A denotes absolute). At 20 kHz, the SPL rises to $\sim$ 100 dBA, and Fig. 3e shows the spectrum for the piezoelectric speaker in the 0–200-kHz interval, as determined from the FFT of the detector voltage waveform. It is immediately evident that the spectrum is almost devoid of harmonic features. The amplitude of the second harmonic ($m=2$) produced by the electromechanical speaker is >40 dB weaker than that of the fundamental at 20 kHz. Beyond *m* = 2, the spectrum recedes into the noise.

The contrast between the microplasma spectra of Fig. 3a–d and the piezoelectric speaker spectrum of Fig. 3e is stark. As noted above, the fundamental (*m* = 1) peak dominates the piezoelectric speaker spectrum and the harmonics are faint or have vanished entirely. This behavior is precisely what one expects for weak nonlinear interactions such as second harmonic generation in crystals at optical frequencies, for example, for which the intensity of the second harmonic wave is often only a few percent of that for the fundamental. In such instances, the amplitude of progressively higher harmonics decays rapidly with order number *m* and the nonlinear conversion mechanism is typically described mathematically by perturbative methods [15]. In the present experiments, however, the plasma nonlinearity is sufficiently strong at acoustic frequencies that the amplitudes of at least two harmonics (2nd and 3rd) are greater than that for the fundamental (cf. Fig. 3b, c). Following these most intense spectral peaks (*m* = 2, 3) lies a region comprising the 5th through the 8th harmonics in which the ultrasound intensities are approximately constant. Beyond *m* = 8, however, the harmonic intensities fall rapidly. For the sake of comparison, panel (f) of Fig. 3 superimposes the piezoelectric transducer spectrum of Fig. 3e with that produced by a microplasma jet array, driven at 25 kHz so as to clearly differentiate the two spectra.

The spectra generated by the microplasma jet arrays in the experiments reported here are remarkably similar to the harmonic spectra produced at optical, vacuum ultraviolet (VUV), and extreme ultraviolet (EUV) frequencies when femtosecond time-scale laser pulses generate rare gas plasmas [16, 17]. Known as high-order harmonic generation (HOHG), this nonlinear optical process produces spectra consisting of harmonics of the fundamental laser frequency in which the intensities of the harmonics fall initially but subsequently remain essentially constant, forming a spectral region known as the “plateau”. Beyond the plateau, referred to as “cutoff”, the harmonic intensity declines rapidly. As Li et al. [18] commented in 1989 regarding HOHG: “... neither the plateau... nor the cutoff at high order can be explained by the traditional theory of nonlinear optics in [a] weak field...” Subsequent theoretical work invoked nonperturbative semiclassical methods to describe the mechanism by which HOHG spectra are produced—namely, electrons driven by the intense optical field [18–20]. Quantum nonperturbative methods were also adopted to interpret HOHG [21, 22]. Similarly, the harmonic spectra of Fig. 3 appear to be generated by a plasma nonlinearity that cannot be described by traditional perturbative theory.

**Fig. 4:**
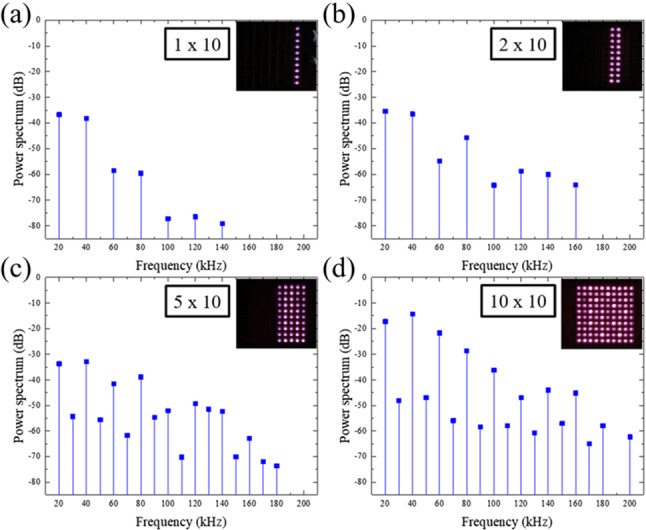
Power spectra in the 0–200-kHz frequency interval for microplasma jet arrays of varying size. Photographs of the arrays in operation are shown at upper right in each panel, and the voltage driving the arrays was fixed at 848 V$_\textrm{RMS}$

**Fig. 5:**
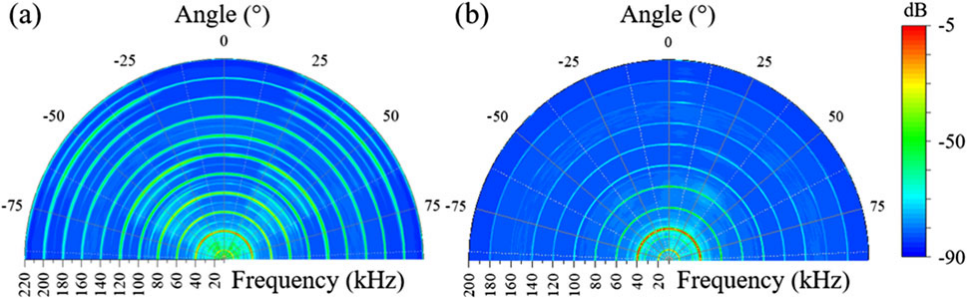
**a** Polar plot (shown in false color) of the dependence of the ultrasound emission spectra generated by a 10 $\times$ 10 array of microplasma jets. The abscissa gives the emission frequency (in kHz), and the color scale at right provides relative intensities. This hemispherical representation assumes the exit plane for the jet array coincides with the abscissa and the microjets are propagating upward; **b** data similar to those of **a** for the static array shown in operation in He in Fig. 1d

We, therefore, interpret the spectra of Fig. 3 as arising from a nonlinearity associated with free plasma electrons and ions oscillating at the driving frequency. Such a dipole radiator, and ensembles of electric dipoles, gives rise to acoustic waves propagating away from the microplasma array. Further support for this conclusion is provided by the data of Figs. 4 and 5, which reveal both spatial and spectral symmetry effects in the emitted acoustic radiation.

The spectra of Fig. 4 demonstrate that one impact of the array geometry is to provide control of the spectrum itself. Panel (a), for example, shows the ultrasound power spectrum generated by a $1\times 10$ array (i.e., a single column of microplasma jets). In this microjet configuration, only integral multiples of the 20 kHz driving frequency (fundamental) are observed, up to *m* = 7, and the harmonic amplitude falls with increasing *m* in a “staircase” fashion. The addition of a second, parallel column of microplasma jets to form a 2 $\times$ 10 array strengthens the higher harmonics ($m=4{-}8$) considerably (by almost 20 dB), as illustrated in Fig. 4b, but only integral harmonics are again observed. However, as the size of the array is extended to 5 $\times$ 10 and 10 $\times$ 10 (Fig. 4c, d, respectively), the fractional harmonics mentioned earlier in connection with Fig. 3d are observed. One concludes that the appearance of fractional harmonics is a symmetry effect associated with the array geometry in combination with a plasma nonlinearity.

The microjet arrays of Figs. 1a, b, 3, and 4 can be regarded as Yagi-Uda phased array antennas, which consist of two or more subwavelength dipoles arranged in parallel [23]. Developed originally for RF frequencies, Yagi-Uda antennas preferentially emit radiation broadside to the array which significantly improves the directionality of the antenna relative to the toroidal pattern emitted by a single, isolated dipole. In the present experiments, each microjet plasma is a self-supporting dipole that is driven by the same time-varying voltage exciting all of the dipoles. A single column of plasma microjets, therefore, constitutes a Yagi-Uda antenna emitting acoustic radiation broadside to the array—i.e., two acoustic waves propagating outwardly in a vertical plane. The two waves generated by each column interfere with each other in much the same way as optical radiation emerging from a linear array of slits. Similar comments could be made for each *row* of microjets in a two-dimensional array except that the outward-propagating acoustic waves lie in a horizontal plane. Consequently, the outward-propagating waves generated by each column or row of microplasmas interfere with those produced by all others.

A by-product of the interference of acoustic waves in arrays of plasma microjets is the observed directionality of the ultrasound emission. Consider, for example, the false color polar plot of the ultrasound emission intensity shown at left in Fig. 5. In this hemispherical representation, the exit plane of the jet array is lying along the abscissa (the emission frequency, labeled in kHz) and the array is oriented such that the plasma jets are propagating upward. Consequently, this graph displays the emission intensity radiated into the angular interval of $-90^\circ \rightarrow +90^\circ$ (with respect to the surface normal of the array), and the color scale at right provides the relative emission intensity, expressed in dB. It is immediately evident that the array does not radiate uniformly but rather emits preferentially into an inverted cone centered at $\sim \pm$ 45$^\circ$ but having a width (cone “thickness”) of ± 20$^\circ$. The observed asymmetry appears to be the result of interference between the broadside emission generated by the rows and columns of plasma-based electric dipoles. As discussed earlier, the rows of plasma microjets generate two outward-propagating waves moving parallel to the exit plane of the jets. These counter-propagating waves propagate parallel to the microjet plasma exit plane, but a component of the waves also propagates away from that surface as well. Similarly, the microplasma columns produce acoustic emission waves propagating vertically, and a portion of both waves propagates outward from the jet exit plane. The vector sum of the wave components propagating parallel to the array exit plane, and those propagating orthogonal to the plane yields resultant waves propagating at angles of ± 45$^\circ$ with respect to the surface normal, thereby forming a cone of acoustic radiation. Note, too, in Fig. 5a the presence of strong emission at the half-harmonic frequencies of 50, 70, and 90 kHz.

**Fig. 6:**
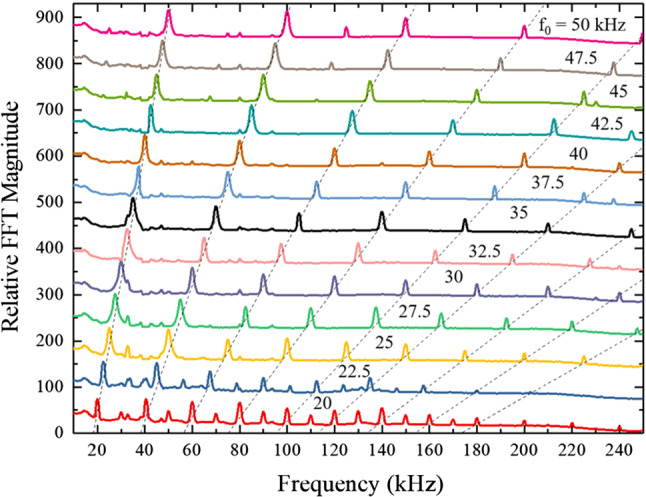
Comparison of the ultrasound emission spectra generated by a $10 \times 10$ plasma jet array at driving frequencies ranging from 20 to 50 kHz. Throughout these experiments, the driving voltage was maintained at 848 V$_\textrm{RMS}$ and, for clarity, the spectra have been displaced vertically from one another

The microjet azimuthal and spectral emission data of Fig. 5a differ considerably from those obtained for the *static* microplasma array, as shown in panel (b) of Fig. 5. In contrast to the jet array, the emission from the static (sealed) microplasma geometry is weaker, more azimuthally uniform and the fractional harmonics have vanished with the exception of emission at 35 kHz and 50 kHz. Figure 6 summarizes the ultrasound emission spectra recorded for jet array driving frequencies ranging from 20 to 50 kHz. The dashed lines in the figure show that the positions of the first nine harmonics follow the driving frequencies as expected but the fractional harmonics weaken considerably with increasing driving frequency. To summarize the results presented in this section, arrays of plasma microjets generate acoustic radiation at both harmonics and half-harmonics of the plasma driving frequency. Furthermore, acoustic emission propagates away from the exit face of the microjet arrays in the form of an inverted cone. The angle of the cone surface with respect to the microjet exit plane surface normal is 45$^\circ$. The ultrasound intensity rises (as would be expected) as the size of a jet array is increased, but the appearance of half-integer harmonics, in particular, has not been observed previously. The contour of the ultrasound spectra closely resembles those observed at optical frequencies by high-order harmonic generation, and the acoustic emission of columns and rows of microjets has been interpreted in terms of Yagi-Uda phased arrays of electric dipoles. Although the physics of ultrasound emission from these microjet arrays is classical, the applications are expected to be significant for imaging, in particular.

## Deep-UV bandpass filters and mirrors fabricated by microplasma-assisted ALD

**Fig. 7:**
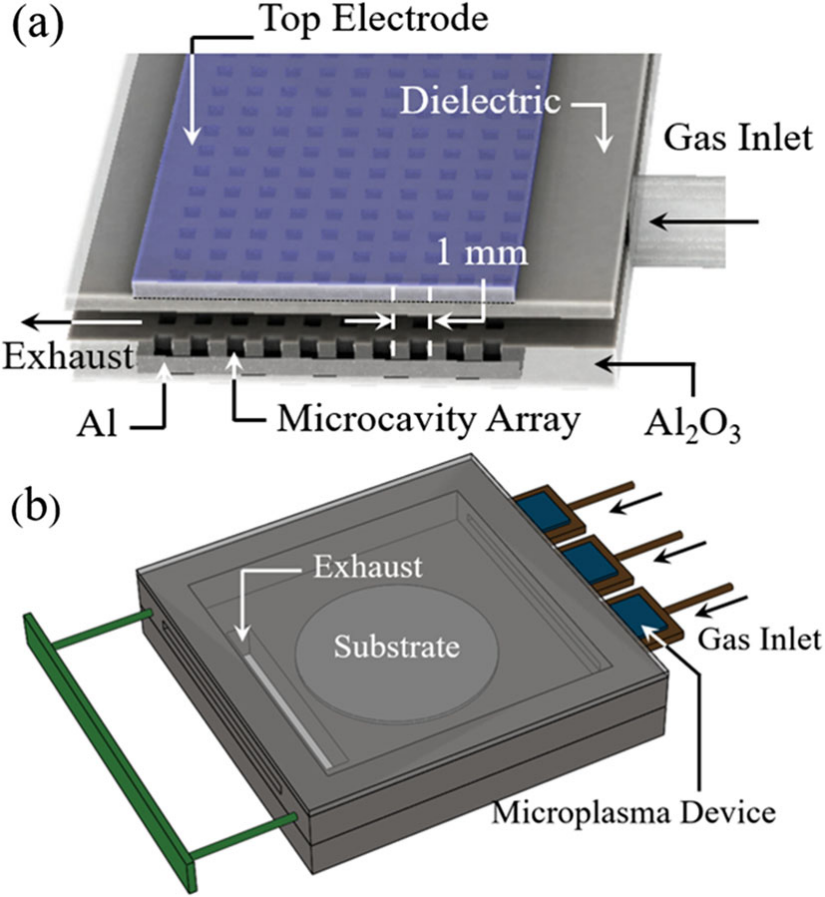
Schematic diagrams of the MALD reactor designs adopted for the fabrication of deep-UV optical structures and components: **a** diagram of the internal structure of the 50 $\times$ 20 microcavity plasma array, fabricated in nanoporous alumina and having one integrated Al electrode. The array pitch (center-to-center spacing of microcavities) is 1 mm; **b** layout of the assembled MALD reactor, showing the precursor inlets at upper right and exhaust exiting at left. The total volume of the reactor is $28 \times 28 \times 1$ cm$^3$

Recently, Kim et al. [24] demonstrated that the introduction of microplasma arrays into atomic layer deposition (ALD) systems reduces the reliance on substrate temperature, thereby allowing dielectric films of high electrical and optical quality to be deposited at temperatures considerably lower than those normally required. Specifically, microplasma arrays provide a compact means for dissociating strongly bound precursors such as O$_2$ and NO but, of equal importance, also produce radicals and excited species capable of favorably altering the chemistry at the interface between the substrate and growing film so as to permit the substrate temperature to be reduced significantly. Experiments conducted at the University of Illinois have deposited both Al$_2$O$_3$ and Ga$_2$O$_3$ films at substrate temperatures of 50 $^\circ$C and room temperature, respectively, by this process known as microplasma-assisted ALD (MALD). Aluminum oxide films 30 nm in thickness, for example, were characterized electrically in metal-oxide/semiconductor capacitors (MOSCAPs) and found to have values of electrical breakdown strength ($E_b$) above 4.1 MV/cm [24]. Another asset of microplasma arrays for film deposition, growth, and etching is their compact size, which allows for multiple arrays to be situated within the reactor and in proximity to one or more substrates. One advantage of positioning microplasmas near a substrate is that short-lived, excited atomic and molecular fragments, produced from a precursor by the microcavity plasma, are able to survive the trip to the substrate before deexcitation occurs and thus contribute beneficially to the surface chemistry. We wish to note that, for the precision deposition of high-quality oxide films, microplasma arrays are an enabling technology because the thermal dissociation of molecular oxygen typically requires substrate temperatures above 1000 $^\circ$C owing to the strength of the O=O bond. Consequently, many ALD systems employ water vapor as the precursor for oxygen in the deposition of oxide films, which generally introduces hydrogen into the film. We report here the fabrication by MALD of multilayer optical bandpass filters and mirrors in the deep-ultraviolet (UV) spectral region. By employing microplasmas to “crack” (i.e., dissociate) O$_2$ without the assistance of high substrate temperatures, alternating layers of ZrO$_2$ and Al$_2$O$_3$ can be grown at temperatures well below those required in the past, thereby allowing for bandpass filters, mirrors, polarizers and other optical and electronic components based on stacks of atomic layers to be realized. A significant benefit of fabricating optical or electronic structures comprising single-atom multilayers at reduced temperatures is minimizing or eliminating interlayer diffusion. Multilayer metal-oxide optical filters designed to have peak transmission near 222 nm in the deep-UV region of the spectrum have been fabricated and characterized. Alternating layers of ZrO$_2$ and Al$_2$O$_3$, each having a thickness in the 20–50 nm range, were grown on quartz and silicon substrates by successively exposing the substrate to the Zr or Al precursor (tetrakis(dimethylamino) zirconium [TDMAZ] or trimethylaluminum [TMA], respectively) and the products of oxygen microplasmas while maintaining the substrate temperature at 300 K. Bandpass filters comprising 7 or 9 cycles of 30 nm-thick ZrO$_2$/50 nm-thick Al$_2$O$_3$ film pairs transmit 80% at 223 nm but only 25% in the 255–260 nm interval. Although the bandpass filters described below were designed expressly for suppressing long wavelength (240–270 nm) radiation emitted by KrCl (222 nm) lamps, the experimental results suggest that the breadth of materials that can be deposited, and optical, electronic, and biomedical devices fabricated, by MALD is extensive. The detailed structure of a microcavity plasma array, and a schematic diagram of the MALD reactor design adopted for the present experiments, are given in panels (a), and (b), respectively, of Fig. 7. The overall volume of the reactor is 28 $\times$ 28 $\times$ 1 cm$^3$, and three arrays of microcavity plasmas were located upstream of the reactor for the purpose of producing oxygen plasma. Each of the two-dimensional microplasma arrays (Fig. 7a, b) comprised 1 mm$^2$ cross-sectional cavities with an intracavity separation (pitch) of 1 mm. The depth of the plenum was increased to 5 mm from 3 mm (in earlier tests) to avoid potential clogging of the Zr or Al precursor lines, and the overall plasma volume (microcavities plus plenum region) for each array is $\sim$ 9 cm$^3$ (6 cm $\times$ 3 cm $\times$ 0.5 cm). The volume of the reactor itself is currently 28 $\times$ 28 $\times$ 1 cm$^3$, which will accommodate substrates as large as 200 mm (8”) in diameter. The three microplasma arrays are located at entrance ports to the reactor, which was found to be optimal for the suppression of corona discharges while maintaining proximity to the substrate. Further details regarding the reactor design and the MALD process can be found in Ref. [24].

Zirconium oxide thin films having thicknesses between 20 nm and 50 nm were deposited on quartz or Si substrates at 300 K by the MALD process in which tetrakis(dimethylamino) zirconium (TDMAZ, 99.99% purity, Strem Chemicals) and oxygen (99.999%) served as the precursors. Because TDMAZ reacts quickly with moisture and is a liquid at room temperature, this precursor was heated to 75 $^\circ$C in a bubbler and research-grade N$_2$ (99.999% purity) served as the carrier gas. Nitrogen also served as the carrier gas for trimethylaluminum (TMA, 98% purity, Strem Chemicals), which has a sufficient vapor pressure at room temperature to not require heating. Mass flow controllers and high-speed on/off valves regulated the flow of precursors and carrier gases into the vertical hot wall reactor. The MALD flow cycle was fully automated and consisted of alternate cycles for the deposition of ZrO$_2$ and Al$_2$O$_3$. The ZrO$_2$ process comprised: (1) a 150 ms dose of TDMAZ, (2) a 60 s nitrogen purge at a flow rate of 50 sccm, (3) 10 s flow of O$_2$ microplasma effluent at a flow rate of 50 sccm, and (4) a final 60 s N$_2$ purge. The corresponding cycle for Al$_2$O$_3$ comprised: (1) a 0.1$-$0.5 s dose of TMA, (2) a 5–10 s N$_2$ purge, (3) 5–10 s flow of O$_2$ microplasma effluent at a flow rate of 50 sccm, and (4) a final 5–10 s N$_2$ purge. During the film deposition process, the N$_2$ carrier gas pressure was maintained at 5 Torr. Operating with such a high carrier gas pressure is possible because of the microplasma arrays. Fabrication of most of the ZrO$_2$/Al$_2$O$_3$ optical structures described below entailed repeated ZrO$_2$ and Al$_2$O$_3$ deposition cycles.

**Fig. 8:**
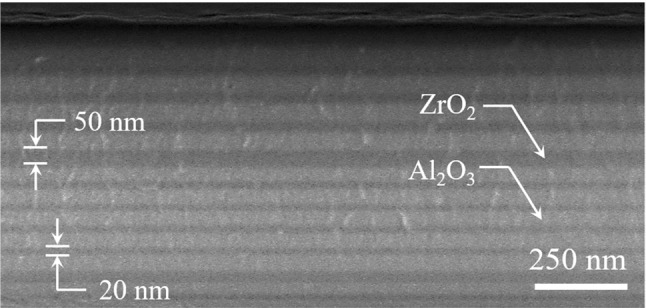
Representative cross-sectional SEM image of a stack of ZrO$_2$ and Al$_2$O$_3$ films, each having a thickness of 20–50 nm and deposited by the MALD process on a Si(100) substrate

**Fig. 9:**
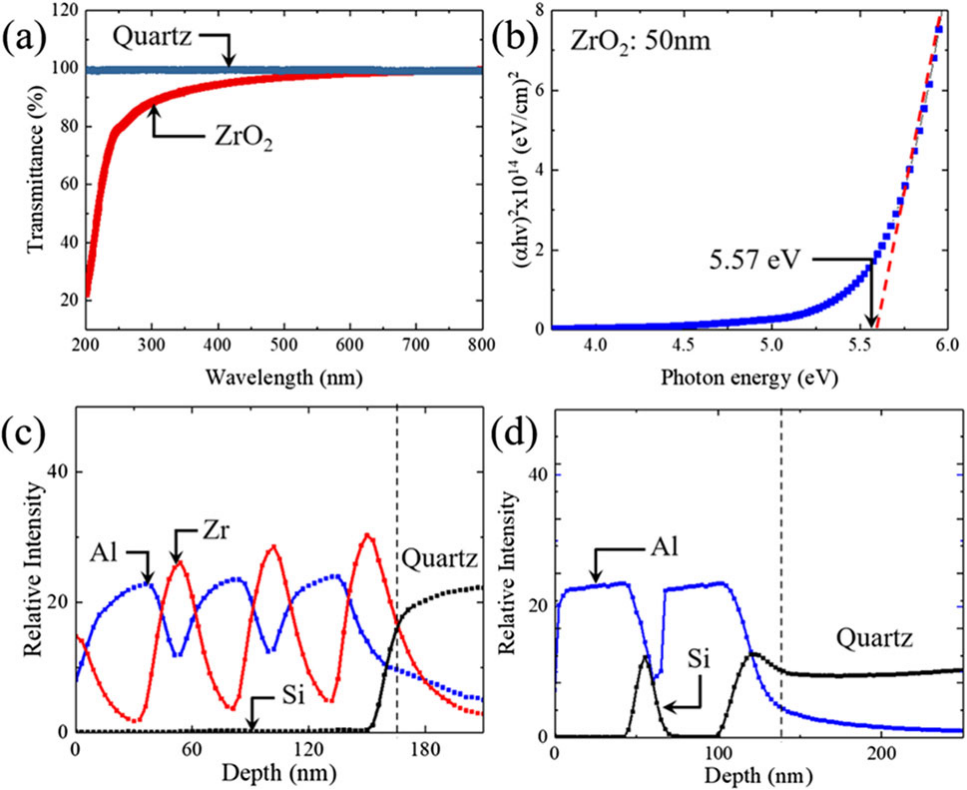
Characterization of the optical transmission and uniformity of thin oxide films deposited by MALD: **a** transmittance spectrum of a 50 nm-thick ZrO$_2$ film deposited on quartz. The black and red curves indicate the transmittance of quartz and the ZrO$_2$ film, respectively. **b** Recasting the data of (**a**) in the form proposed by Tauc [25] so as to determine the optical bandgap of the ZrO$_2$ thin film; **c** elemental depth profiles for Al, Zr, and Si acquired by TOF-SIMs for a 3-cycle ZrO$_2$/Al$_2$O$_3$ film stack deposited by MALD onto a quartz substrate. The red, blue, and black curves represent Zr, Al and Si, respectively; **d** TOF-SIMs depth profiles for Al and Si in a simple Al$_2$O$_3$/SiO$_2$ thin film stack deposited by an electron beam evaporator

Figure 8 is a scanning electron micrograph (SEM) image of a stack of ZrO$_2$ and Al$_2$O$_3$ films deposited on a Si(100) substrate by MALD. The thickness of two of the layers is indicated in Fig. 8, and all of the layers have thicknesses in the range of 20–50 nm. The optical quality of the ZrO$_2$ films, as well as the uniformity and composition of both the ZrO$_2$ and Al$_2$O$_3$ layers, was characterized by optical transmission measurements and secondary ion mass spectrometry (SIMS), and the results of several tests are presented in Fig. 9. The measured optical transmission spectrum of a 50-nm-thick ZrO$_2$ film, presented in Fig. 9a, shows evidence of absorption for wavelengths below $\sim$ 450 nm but the onset of rapidly falling transmission at $\sim$ 250 nm in the UV region. The spectrum of panel (a) of Fig. 9 can be recast in the form proposed by Tauc [25] and doing so yields the blue curve (Tauc plot) in the graph of Fig. 9b for which the abscissa is the photon energy. Extrapolating the asymptotic, dashed red line of Fig. 9b indicates that the direct bandgap energy ($E_g$) of the ZrO$_2$ films deposited by MALD is 5.57 eV. Representative results for time-of-flight SIMS (TOF-SIMS) analysis of several ZrO$_2$/Al$_2$O$_3$ and Al$_2$O$_3$/SiO$_2$ film stacks are shown in Fig. 9c, d, respectively. In panel (c) of Fig. 9, elemental depth profiles for Zr, Al, and Si measured by TOF-SIMS for a 3-cycle ZrO$_2$/Al$_2$O$_3$ stack illustrate the uniformity of the layers. Much of the gradual tapering at the front and back edges of the individual layers, and the apparent “bleeding” of zirconium into the quartz substrate is an artifact arising from the limited resolution of the SIMS instrument. For the sake of comparison, Al$_2$O$_3$/SiO$_2$ film stacks deposited by an electron-beam evaporator were also examined by TOF-SIMS and the representative data of Fig. 9d exhibits the same tapering of the depth profiles observed for ZrO$_2$/Al$_2$O$_3$ film stacks, as expected. One concludes, therefore, that the spatial and compositional quality of the MALD deposited films is high.

Given the precision film deposition capability described above, several multilayer ZrO$_2$/Al$_2$O$_3$ bandpass filter structures were designed for the 200–300 nm wavelength region with commercially available software. The motivation for these calculations was the development of bandpass filters intended to reject a portion of the longer-wavelength UV radiation (i.e., wavelengths $>230$ nm) emitted by deep-UV krypton chloride (KrCl) lamps. Commercially available KrCl (222 nm) lamps have been applied successfully during the COVID-19 pandemic to disinfecting both the air and surfaces in public spaces such as those in restaurants and public transportation [26]. Unlike conventional UV sources such as the 254 nm resonance line of Hg, emitted by “germicidal” lamps, multiple studies have shown that humans can safely be present when 222-nm lamps are operating because such deep-UV photons are absorbed in the outermost layer of human skin and, therefore, are unable to reach DNA. However, although >95% of the radiation emitted by the KrCl molecule lies within a few nanometers of 222 nm, a few percent of the radiated power extends in wavelength past 230 nm to beyond 250 nm. Despite its low intensity, this longer-wavelength emission is of medical concern and bandpass filters designed to absorb or otherwise reject this radiation are required. The ZrO$_2$/Al$_2$O$_3$ structures reported here represent the initial attempt to design a new class of such optical filters.

**Fig. 10:**
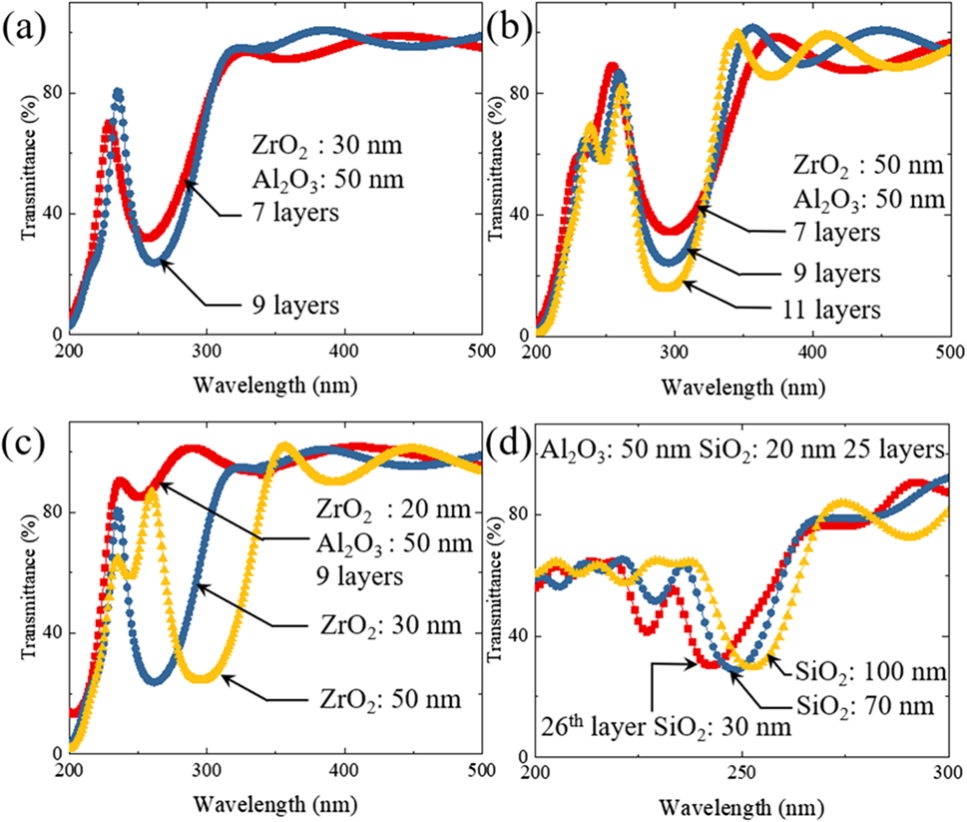
Transmission spectra recorded in the 200–500 nm wavelength range for several ZrO$_2$/Al$_2$O$_3$ and SiO$_2$/Al$_2$O$_3$ bandpass/bandstop filters fabricated on quartz by MALD and e-beam evaporation, respectively: **a** bandpass filter comprising seven (red curve) or nine (blue) film cycles, each comprising alternating 30-nm-thick ZrO$_2$ and 50-nm-thick Al$_2$O$_3$ thin films; **b** impact on the transmission spectra of 50 nm ZrO$_2$/50 nm Al$_2$O$_3$ film stacks of increasing the number of ZrO$_2$/Al$_2$O$_3$ cycles from 7 to 11; **c** dependence of the transmission spectrum of ZrO$_2$/50-nm-thick Al$_2$O$_3$ thin film stacks on increasing the ZrO$_2$ film thickness from 20 to 50 nm. Each of the stacks has 9 ZrO$_2$/Al$_2$O$_3$ film cycles; **d** transmission spectrum for a bandstop filter comprising 25 cycles of alternating 20-nm-thick SiO$_2$ and 50-nm-thick Al$_2$O$_3$ thin films. The three curves shown correspond to different thicknesses of the 26th (SiO$_2$) film, and all films were deposited by electron beam evaporation

Figure 10 presents several series of optical transmission spectra that were recorded with a Cary 5 G spectrophotometer over the 200–500 nm wavelength interval for various designs of ZrO$_2$/Al$_2$O$_3$ and SiO$_2$/Al$_2$O$_3$ bandpass or bandstop filters deposited on quartz substrates. The former of these filters were fabricated by MALD and the latter by electron beam evaporation. The highest degree of discrimination obtained to date between 222 nm radiation and the longer-wavelength emission of the KrCl molecule was observed with the ZrO$_2$/Al$_2$O$_3$ multilayer filters represented by Fig. 10a. In fabricating this set of filters by MALD, the thickness of the alternating ZrO$_2$ and Al$_2$O$_3$ films was set at 30 nm and 50 nm, respectively. Spectra are presented for filters comprising 7 and 9 ZrO$_2$/Al$_2$O$_3$ film cycles and, as expected, increasing the number of such ZrO$_2$/Al$_2$O$_3$ cycles sharpens the desired peak observed in the $\sim$ 225–230 nm range. Although the addition of two layers to the 7-layer structure also moves the deep-UV peak to $\sim$ 235 nm, which is a few nm beyond the target of 222 nm, the transmission of the 9-layer filter in the critical 240–290 nm region is reduced by as much as 8% relative to that for the 7-layer structure. That is, peak transmission for the 7-cycle filter is 70% at 232 nm but 30% at 255 nm, whereas maximum transmission for the 9-layer structure is 80% at 235 nm and 23% at 260 nm. Note that the maximum transmission of the filter below 230 nm is constrained solely by the ZrO$_2$ films (cf. Figure 9a). If the thickness of both the ZrO$_2$ and Al$_2$O$_3$ films is now fixed at 50 nm but the number of ZrO$_2$/Al$_2$O$_3$ film cycles is increased incrementally from 7 to 11, one observes the transmission spectra of Fig. 10b. Increasing the thickness of the ZrO$_2$ films in the structure has the undesirable effect of creating a second transmission peak in the deep-UV region at $\sim 250$ nm. Once again, increasing the number of ZrO$_2$/Al$_2$O$_3$ film cycles suppresses transmission at longer wavelengths, but this dip has now moved to longer wavelengths (285–305 nm). In panel (c) of Fig. 10, the impact on the filter transmission of varying the thickness of the ZrO$_2$ films is shown. If this thickness is reduced to 20 nm (red curve, Fig. 10c), the bandpass nature of the ZrO$_2$/Al$_2$O$_3$ structure is lost, whereas extending the thickness to 50 nm (yellow curve) yields the transmission spectrum discussed in connection with panel (b) of Fig. 10. A second approach to preferentially suppressing radiation in the $\sim$ 239–290 nm region was pursued with SiO$_2$/Al$_2$O$_3$ multilayer structures deposited by electron-beam evaporation. One example is that of Fig. 10d which displays transmission spectra comprising 26 SiO$_2$/Al$_2$O$_3$ film cycles. For the first 25 cycles, the SiO$_2$ and Al$_2$O$_3$ film thicknesses were held constant at 20 and 50 nm, respectively. However, a 26th layer was added in which the Al$_2$O$_3$ layer thickness was maintained at 50 nm but the thickness of the SiO$_2$ films was varied in increments, from 30 nm to 100 nm. Although these SiO$_2$/Al$_2$O$_3$ structures were successful in attenuating radiation in the 235–255-nm spectral interval to $\sim$ 30%, peak transmission at 222 nm is only $\sim$ 60% and two sets of SiO$_2$/Al$_2$O$_3$ multilayer structures are necessary to yield a bandstop filter capable of covering more than 10–15 nm.

**Fig. 11:**
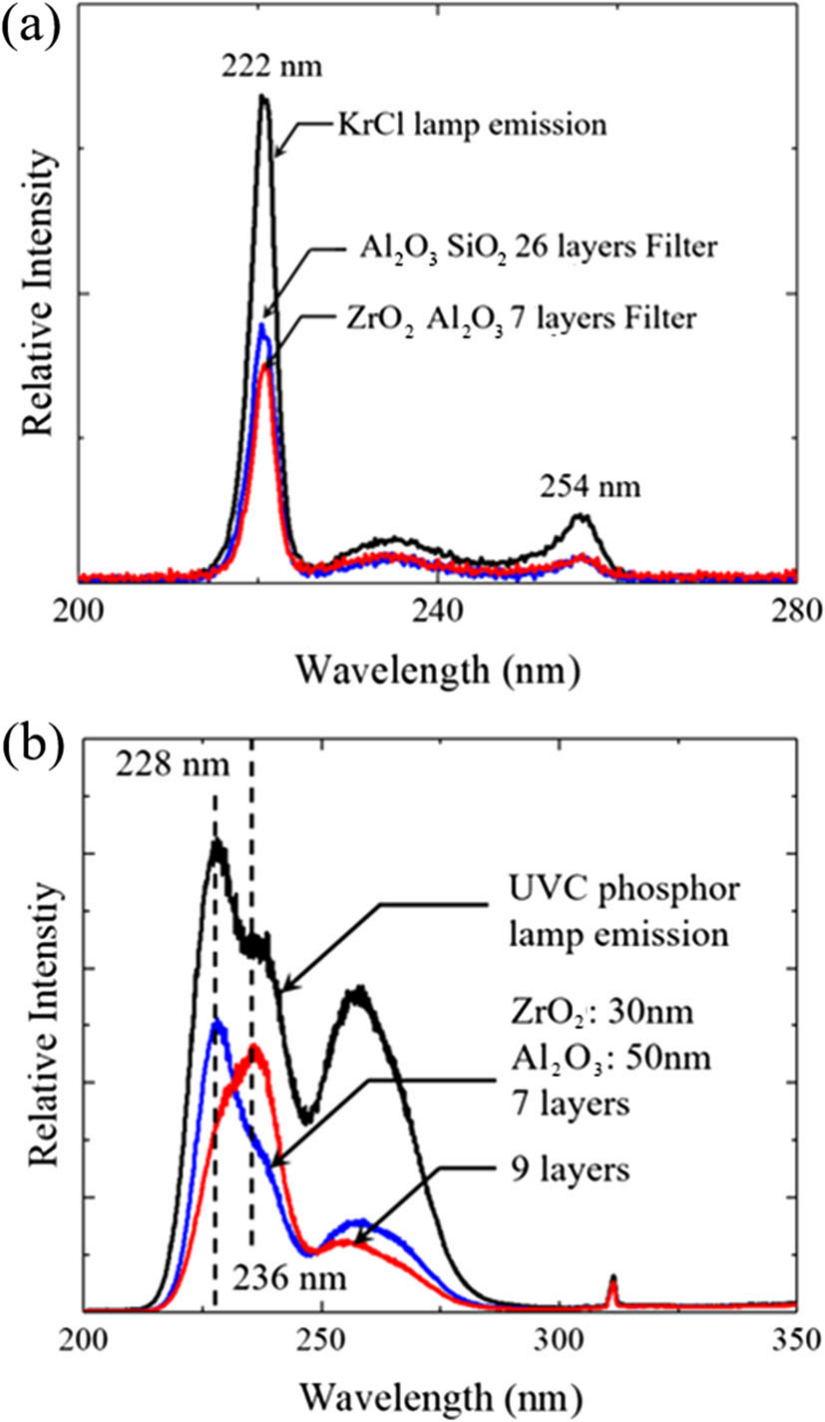
Transmission spectra illustrating the influence of ZrO$_2$/Al$_2$O$_3$ multilayer bandpass filters on the output spectrum of deep-UV microplasma-based lamps: **a** emission spectrum of a commercial KrCl lamp in the absence of any spectral filtering (black trace), showing peak emission at 222 nm. When the 7-layer ZrO$_2$/Al$_2$O$_3$ bandpass filter of Fig. 10a is placed on the exit window of the 222 nm lamp, the spectrum shown in red is observed; **b** similar spectra recorded for a deep-UV lamp comprising a UV phosphor applied to the inner surface of the exit window for a flat, Xe$_2$ (172 nm) microplasma lamp. The emission spectrum for the lamp itself is shown in black whereas the effect of adding the 7-layer or 9-layer ZrO$_2$/Al$_2$O$_3$ bandpass filters of Fig. 10a are represented by the blue and red curves, respectively

Figure 11 illustrates modifying the emission spectrum of a deep-UV microplasma lamp with one of the ZrO$_2$/Al$_2$O$_3$ multilayer bandpass filters of Fig. 10. Panel (a) of Fig. 11, for example, is the emission spectrum for a commercially available KrCl lamp in the absence of any spectral filtering (black curve). When the 7-layer ZrO$_2$/Al$_2$O$_3$ bandpass filter of Fig. 10a is placed onto the exit window of this lamp, however, the recorded spectrum is indicated by the red curve. Note that the KrCl lamp spectrum has been attenuated throughout, but not uniformly. In other words, the KrCl emission peak at 222 nm has been suppressed by approximately 55% by the insertion of the ZrO$_2$/Al$_2$O$_3$ bandpass filter into the optical path but the broad continuum having a maximum local intensity at $\sim 254$ nm has been attenuated more strongly (by $\sim$ 63%). As noted earlier, the suppression of approximately a factor of two below 230 nm is attributed solely to ZrO$_2$ film transmission in this region (cf. Figure 9a). It is expected that optimization of the ZrO$_2$ film thickness in the range of 20–30 nm (Fig. 10c), and the number of film layers/cycles will improve the performance of ZrO$_2$/Al$_2$O$_3$ bandpass filters for the purpose of preferentially suppressing radiation beyond 230 nm. Similar spectra were acquired for a second deep-UV lamp consisting of a flat Xe$_2$ microplasma lamp [27] into which a phosphor coating has been applied on the interior surface of the fused silica exit window. The black curve of Fig. 11b is the spectrum of the lamp without the presence of a bandpass filter, whereas the blue and red traces indicate the spectra observed when either the 7-layer or 9-layer ZrO$_2$/Al$_2$O$_3$ filters, respectively, was placed at the exit window of the lamp. It is evident that the filters have strongly attenuated emission in the spectral region beyond $\sim$ 236 nm.

## Summary and conclusions

Over the past 2.5 decades, considerable progress in elucidating the physics of microplasmas, and pursuing their applications, has been made. Two recently developed applications, the efficient generation and control of ultrasound emission in the 20–240 kHz region and the fabrication of precision, multilayer optical components by microplasma-assisted ALD (MALD), have been described. It has undoubtedly not escaped the reader’s attention that the electromagnetic frequency ranges represented by the two applications presented here differ by almost 10 orders of magnitude (i.e., hundreds of kHz $\rightarrow$ deep-UV, 10$^{15}$ Hz). Such a span reflects the unique characteristics of low temperature plasmas having sub-mm dimensions, and the demonstrated ability to confine them to precisely determined cavities. Professor Kurt Becker foresaw many of the capabilities that have been demonstrated since the mid-1990 s and is deservedly viewed universally as a pioneer of this exciting field.

## Data Availability

This manuscript has no associated data or the data will not be deposited. [Authors’ comment: The datasets generated during and/or analyzed during the current study are available from the corresponding author upon reasonable request.]
